# The effect of virtual reality applications on the anxiety levels of emergency department physicians during 24-h shifts

**DOI:** 10.1007/s11739-025-03947-x

**Published:** 2025-04-23

**Authors:** Sinan Paslı, Nurullah Samet Yılmaz, Esma Nilay Kırımlı, Melih İmamoğlu, Muhammet Fatih Beşer, Abdul Samet Şahin, Metin Yadigaroğlu

**Affiliations:** 1 School of Medicine, Department of Emergency Medicine, Karadeniz Technical University, 61080 Trabzon, Turkey; 2 Emergency Service, Nizip State Hospital, Gaziantep, Turkey; 3 Business and Decision Life Sciences, Brussels, Belgium; 4 School of Medicine, Department of Emergency Medicine, Samsun University, Samsun, Turkey

**Keywords:** Anxiety, Emergency resident, Stress, Virtual reality, VR, Well being

## Abstract

This study aims to investigate the effects of virtual reality (VR) interventions on the state anxiety levels of residents working 24-h shifts in an emergency department. This study utilizes a single-center, simulation-based, quasi-experimental design. In the study's first phase, participants' anxiety levels were measured using the STAI-I scale during their shifts, between 7:00 PM and 10:00 PM (Pre-Rest) and after a 10-min rest, a second measurement was performed (Post-Rest). In the second phase, anxiety levels were measured during the same time interval in their next shift (pre-VR), followed by another evaluation after 10 min of using a VR headset (Post-VR). The effects of rest and VR use on state anxiety levels compared. A total of 26 emergency medicine residents participated in the study. The mean age of the participants was 29 ± 2.6 years, with an equal gender distribution. The median pre-rest anxiety score was 46 (28–68), while the post-rest median was 43 (22–62), with a statistically significant difference (*p* = 0.02). The median anxiety score before VR use was 47 (25–73), and after VR use, it decreased to 30 (20–73), which was significant (*p* = 0.001). When comparing the changes in anxiety levels after rest and VR use, the median Δanxiety rest was 4.5 (− 17–25), whereas the median Δanxiety VR was 11.5 (− 21–47) (*p* = 0.017). Based on our study's results, a 10-min VR session during a 24-h shift reduces emergency physicians' state anxiety levels and is more effective than a rest break.

**Graphical Abstract:**

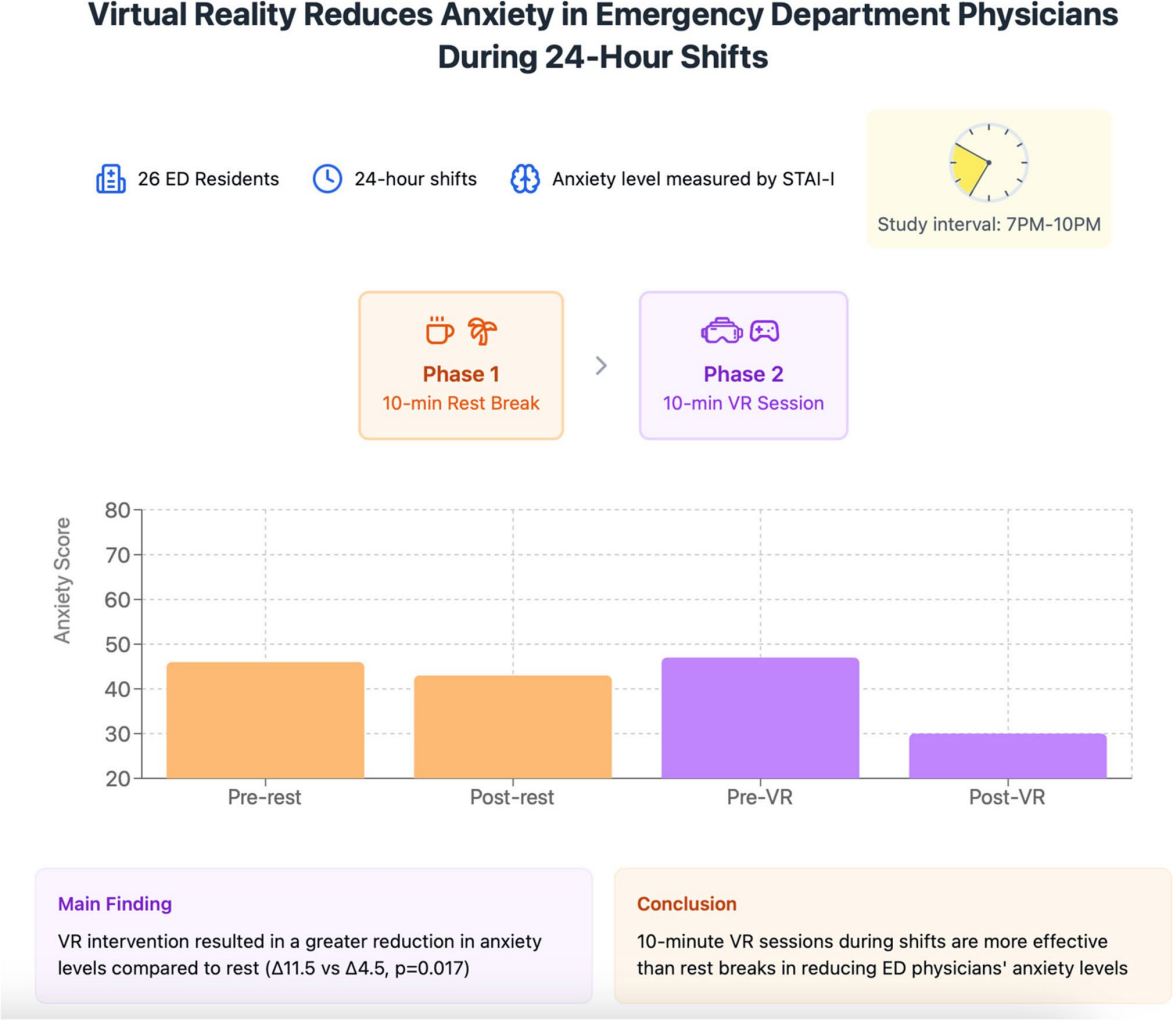

**Supplementary Information:**

The online version contains supplementary material available at 10.1007/s11739-025-03947-x.

## Introduction

In modern healthcare systems, emergency departments (EDs) are one of the most intensive units to which diverse and critical patient groups are admitted. The work schedule of ED staff, particularly physicians, differs markedly from that of their colleagues in other hospital departments. ED personnel is often required to work prolonged hours during day or night shifts, disrupting the natural circadian rhythm [1]. The unnatural disruption of the circadian rhythm places considerable physiological stress on the body, contributing to elevated stress levels. According to the literature, night shift workers face an increased risk of developing chronic conditions such as diabetes, cardiovascular diseases, and depression [2]. Moreover, the frequent lack of resources in EDs forces physicians to exert additional effort, thus increasing both physical and mental strain and exacerbating work-related stress. In addition, the high number of patients, the occurrence of adverse events (medical errors, complications, or workplace violence), and long patient waiting times are recognized as important stress factors for physicians [3]. Numerous studies have also indicated a correlation between extended work hours and adverse outcomes such as increased fatigue and mood disturbances [4, 5]. Additionally, prolonged working hours have been associated with a higher prevalence of depression and anxiety disorders among both men and women [6].

Given that EDs are constantly surrounded by visual, auditory, and tactile stressors, there is a pressing need for innovative solutions to address these challenges and mitigate physician stress. Virtual reality (VR) is a real or simulated environment that provides an immersive audiovisual experience, giving individuals the sensation of physical presence in a location different from their actual surroundings [7]. VR is considered an alternative response of technology to the state of consciousness and has proven useful in improving psychological health by altering perception and resulting behaviour [8]. Integrating VR with exercise creates an immersive experience, allowing users to engage with environments that simulate familiar, novel, or beneficial audio-visual stimuli [9]. This immersive experience contributes to reducing anxiety, stress, and depression, promoting mental well-being through enhanced engagement and altered perception [8].

Most studies on using VR applications in healthcare settings, such as perioperative anxiolysis, managing distress, non-pharmacological sedation, and analgesia for cancer patients, have predominantly focused on patient-centered outcomes [10–13]. The literature contains limited studies focused on healthcare workers, highlighting a gap in research on the potential benefits of VR applications for this population. The aim of this study was to evaluate the effect of VR use on the anxiety levels of residents working 24-h shifts in an ED.

## Methods

### Study design and population

This study utilizes a single-center, simulation-based, quasi-experimental design and was conducted from January 1, 2024, to January 31, 2024. The study population consisted of all emergency medicine residents working in 24-h shifts in a tertiary ED and volunteering to participate in the study. A known history of epilepsy, significant discomfort or side effects related to VR goggles (headache, nausea, dizziness), use of spectacles (due to the physical limitations of the Oculus Quest 2 VR headset, which could not comfortably accommodate standard eyeglasses, and lacks adjustable diopters) and a known diagnosis of psychiatric illness were determined as exclusion criteria.

This study was carried out in strict compliance with the principles of the Declaration of Helsinki following the approval of the Local Scientific Research Ethics Committee (Reference number: 2023/183).

### Facility layout and operational flow of the emergency department

The ED where the study was conducted is a tertiary ED, recognized as the most extensive and best-equipped facility in the region. It is organized into four distinct areas: green, yellow, trauma, and red zones. A resident physician is assigned to a 24-h shift in each of these areas. Additionally, a senior resident oversees operations coordination across all areas and manages communications with emergency medical services. The daily patient volume of ED is approximately 300.

### Data collection

In the first phase of the study, participants' anxiety levels were measured using the State-Trait Anxiety Inventory (STAI-I) scale during a shift between 7.00 pm and 10.00 pm (pre-rest level) [14]. Following this, participants took a 10-min free resting period, after which their anxiety levels were measured again (post-rest level). Participants were free to do any activity during the rest; they were not restricted to a specific activity.

**Fig. 1:**
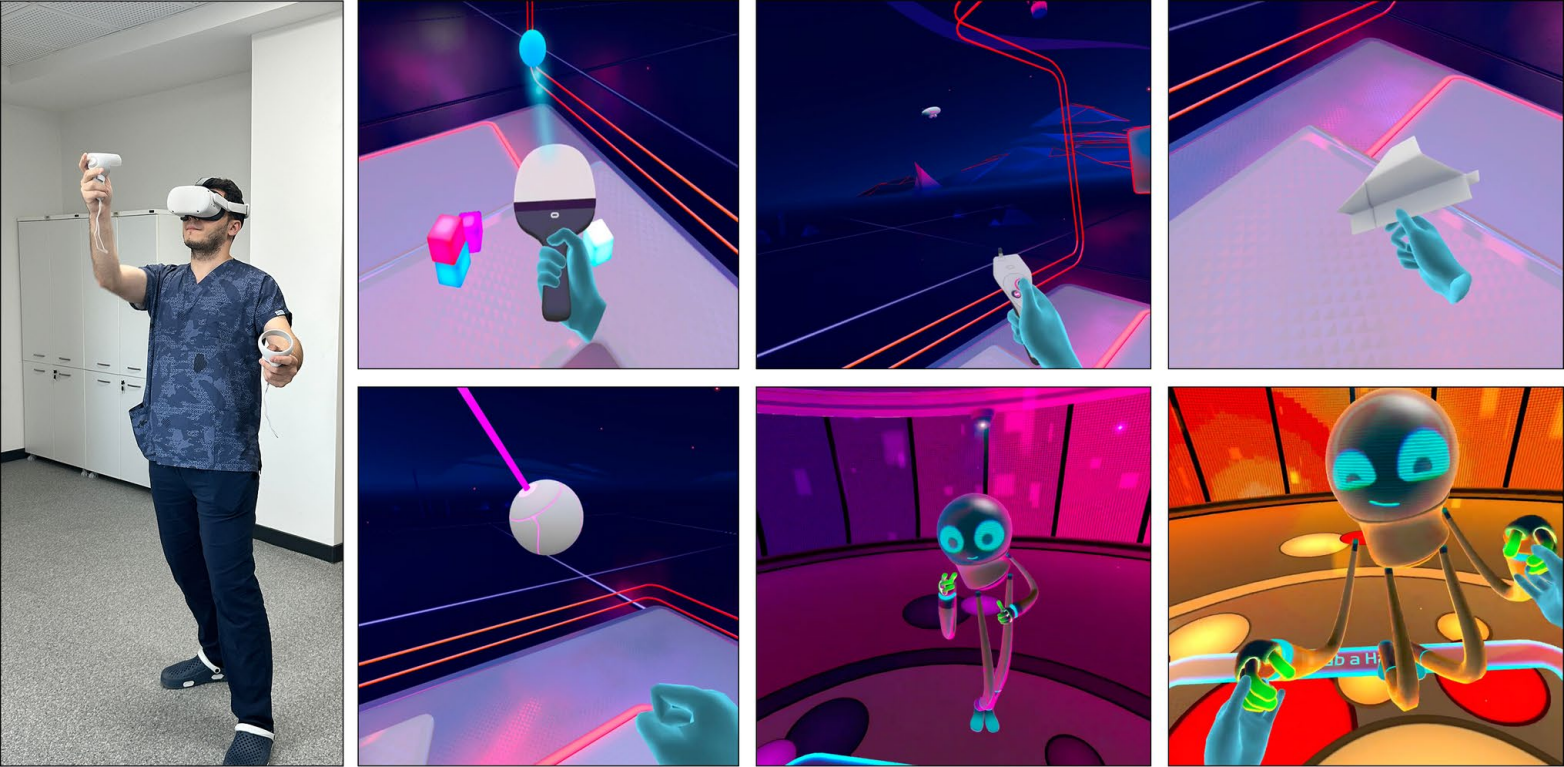
Participant doing various activities with VR headset

In the second phase, anxiety levels were measured during the same time interval in another (next) shift (pre-VR level). Afterward, participants used the “First Steps” application on the Oculus Quest 2 VR Headset (Meta Platforms, Inc., Menlo Park, CA, USA) for 10 min, and their anxiety levels were measured again (post-VR level) [15]. In this application, participants engaged in various activities while familiarizing themselves with the VR goggles. These activities included flying a paper airplane, piloting a blimp, playing tennis, punching a ball, dancing, and more (Fig. 1) (Supplemental video S1).

The 7:00 pm–10:00 pm time interval was selected as it represents the busiest period in the ED. All participants were enrolled in the study within the same month to minimize the impact of seasonal or periodic differences on ED dynamics.

The State-Trait Anxiety Inventory (STAI-I) scale, used to assess anxiety levels, was digitized and transferred to Google Forms. It was then shared with participants via a WhatsApp link sent to their mobile phones (Appendix 1). A second survey, which was designed by the authors, was also distributed via Google Forms to gather participants' opinions and experiences regarding the VR intervention. This form employed a 5-point Likert scale, where 1 indicated negative or disagreement and 5 indicated positive or agreement (Appendix 2). All collected data were transferred to the Microsoft Excel program for analysis.

### Statistical analysis

The collected data were analyzed using Jamovi software (version 2.4.12) with a 95% confidence level. The anxiety levels of resident physicians working in the ED were compared before and after rest, as well as before and after the VR intervention. Subgroup analyses were performed based on gender and experience (less than two years vs. more than two years). Additionally, anxiety change after rest (Δ anxiety rest) and after VR use (Δ anxiety VR) were calculated and compared. To determine the appropriate analysis method, the Shapiro-Wilks test was performed to assess whether the data followed a normal distribution. Descriptive statistics were reported as mean and standard deviation for normally distributed numerical data, median (minimum–maximum) for non-normally distributed data, and frequencies (n) and percentages (%) for categorical data. The Wilcoxon test was used for dependent numerical data for pairwise group comparisons, while the Student's t-test was employed for independent numerical data. A p-value of less than 0.05 was considered statistically significant in all statistical analyses.

## Results

A total of 26 emergency medicine residents participated in the study. The mean age of the participants was 29 ± 2.6 years, with an equal gender distribution. Of the participants, 42% (*n* = 11) had two years or less of experience, while 58% (*n* = 15) had more than two years of experience. The median number of monthly shifts per resident was 8 (7–10) (Table 1).

**Table 1:** Demographic characteristics of participants

Gender	*N* (%)	Age (mean ± sd)
Male	13 (50)	28.8 ± 2.8
Female	13 (50)	29.7 ± 2.3
Years of experience		
≤ 2 years	11 (42)	
> 2 years	15 (58)	
Number of shifts/month		
7	11(42)	
8	5 (19)	
9	3 (12)	
10	7 (27)	
Previous VR experience		
Yes	1 (4)	
No	25 (96)	

**Fig. 2:**
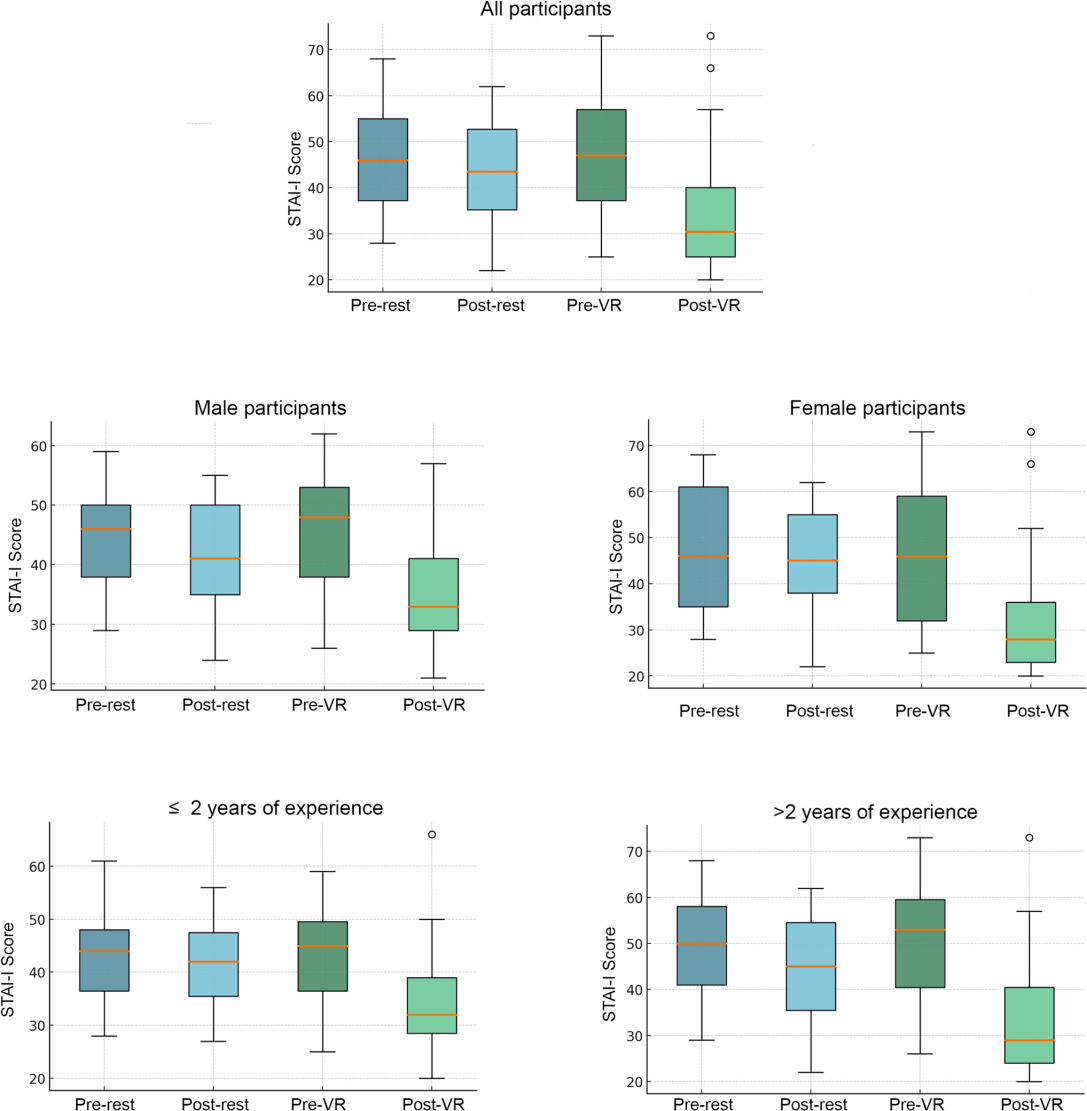
Distribution of STAI-I scores among emergency medicine residents

In the evaluation of anxiety levels during shifts, the median pre-rest anxiety score was 46 (28–68), which decreased to 43 (22–62) post-rest, and the difference was statistically significant (*p* = 0.02). When examining the effect of VR use on anxiety levels, the median anxiety score before VR use was 47 (25–73), which decreased to 30 (20–73) after VR use, with the change being statistically significant (*p* = 0.001) (Table 2) (Fig. 2).

**Table 2:** Comparison of STAI-I scores between “pre-rest”, “post-rest” and “pre-VR”, “post-VR” groups

	^a^Pre-rest median (min–max)	^b^Post-rest median (min–max)	^c^Pre-VR median (min–max)	^d^Post-VR median (min–max)	*p* values
Male	46 (29–59)	41 (24–55)	48 (26–62)	33 (21–57)	^*^P^*a*−*b*^ = 0.021 ^*^P^*c*−*d*^ = 0.011
Female	46 (28–68)	45 (22–62)	46 (25–73)	28 (20–73)	^*^P^*c*−*d*^ = 0.041
≤ 2 years	44 (28–61)	42 (27–56)	45 (25–59)	32 (20–66)	
> 2 years	50 (29–68)	45 (22–62)	53 (26–73)	29 (20–73)	^*^P^*c*−*d*^ = 0.004 ^*^P^*a*−*b*^ = 0.026
Total	46 (28–68)	43 (22–62)	47 (25–73)	30 (20–73)	*P^*a*−*b*^ = 0.022 *P^*c*−*d*^ = 0.001 ^+^*P*^*b*−*d*^ = 0.03
Δ anxiety score	4.5 (− 17–25)		11.5 (-21–47)		^+^*P* = 0.017

STAI: State-Trait Anxiety Inventory

^*^Wilcoxon signed-rank test

+independent two-sample *t*-test

**Fig. 3:**
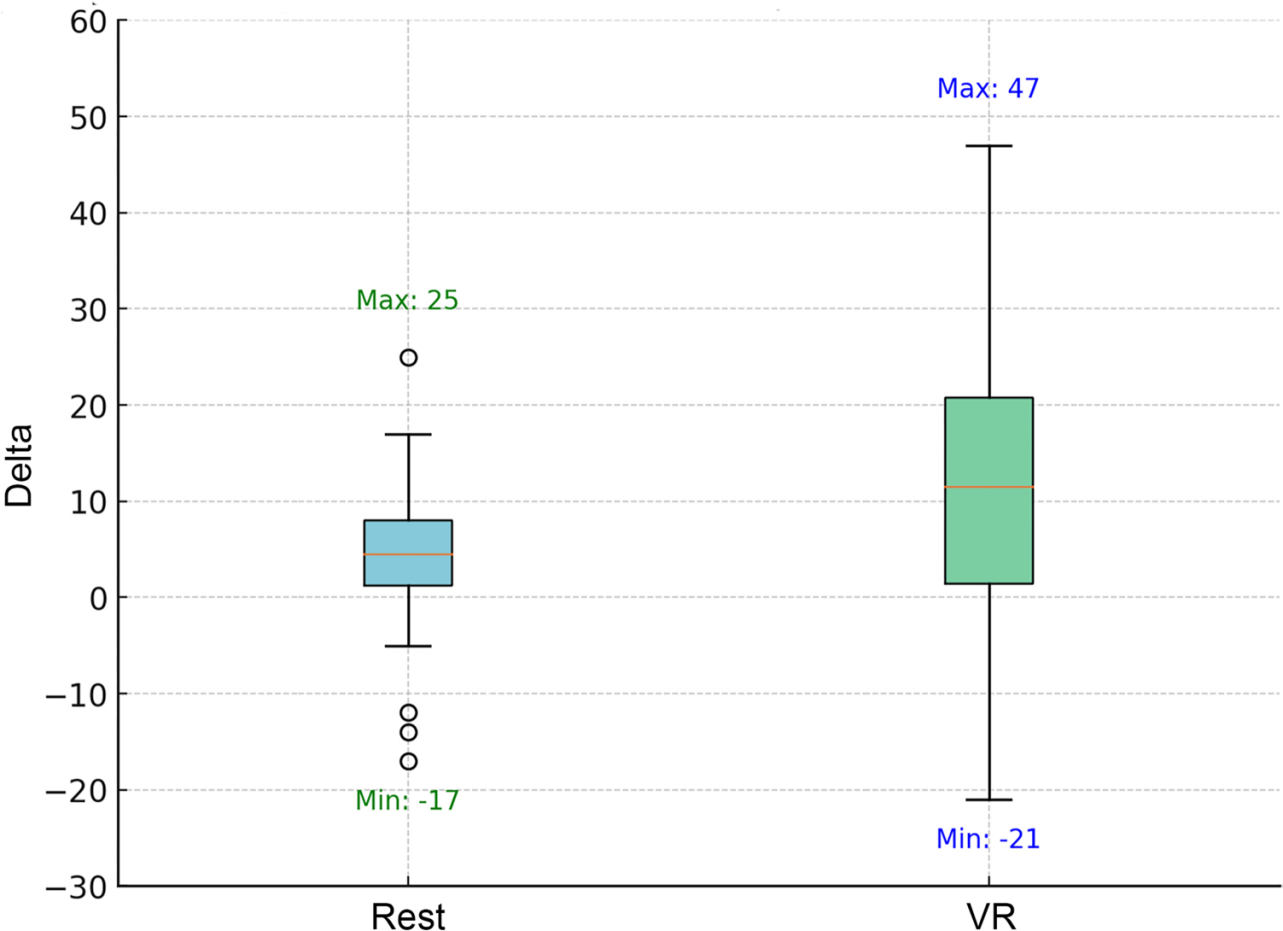
Boxplot comparison of delta anxiety values following VR use and rest

When the changes in anxiety levels following the rest and VR use were compared, the median Δ anxiety rest was 4.5 (range: 17–25), while the median Δ anxiety VR was 11.5 (range: 21–47). This analysis indicated that VR use during the shift led to a significantly greater reduction in anxiety levels compared to the rest (*p* = 0.017) (Fig. 3).

**Fig. 4:**
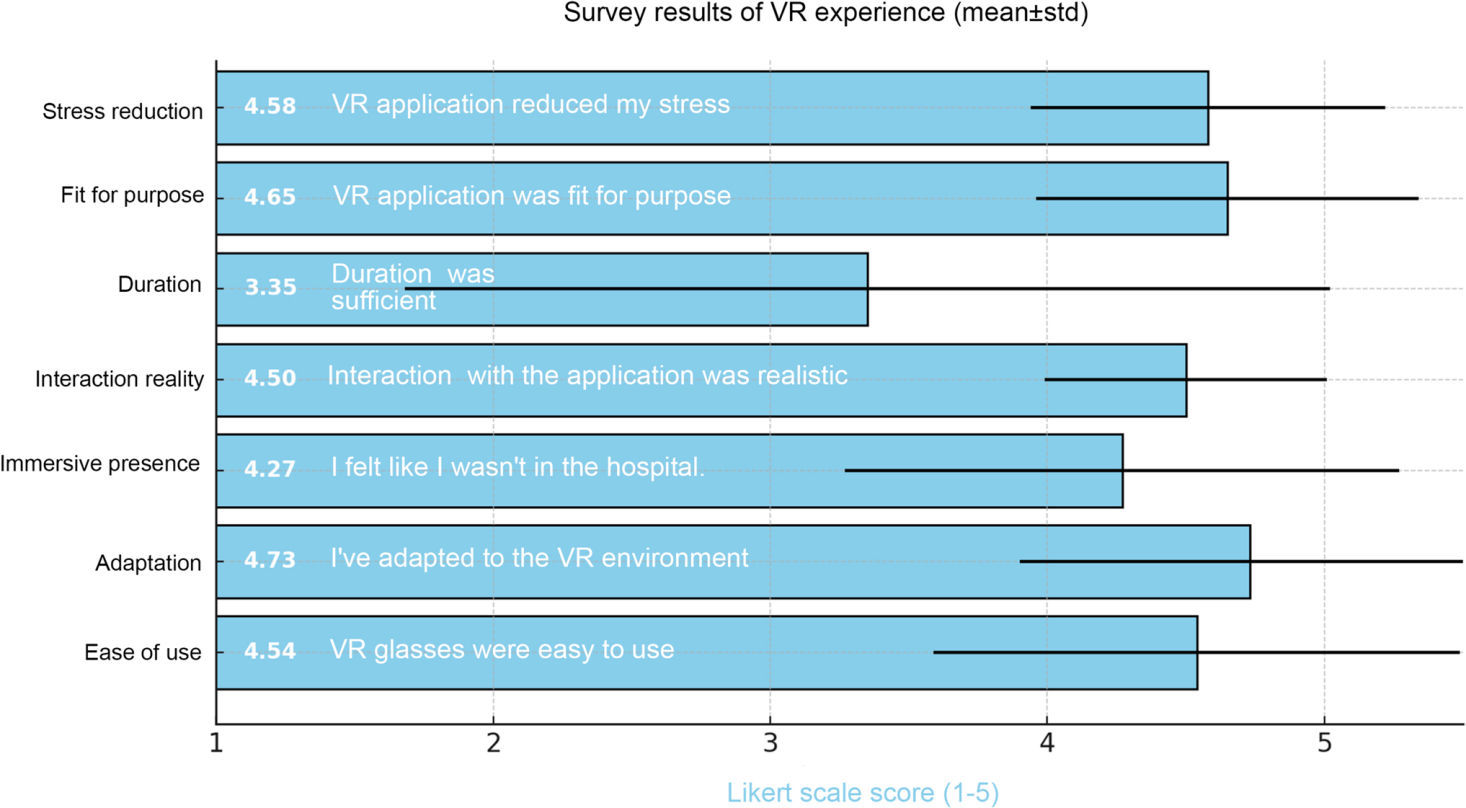
Evaluation of participants’ feedback on VR application through a survey

In this study, participants' experiences with the VR application and any side effects encountered were assessed through a separate survey. According to the survey results, 96% of the participants reported that they had never used VR before. It was observed that participants generally found the VR goggles easy to use (mean:4.54 ± 0.95) and adapted quickly to the virtual environment (mean:4.73 ± 0.83). Most participants reported feeling like they were in a completely different place from the hospital while using the VR environment (mean:4.27 ± 1.00) and found the interactions within the application to be highly realistic (mean:4.50 ± 0.51). Although there were differing opinions regarding the adequacy of the application duration (mean:3.35 ± 1.67), participants generally agreed that the application was appropriate for its intended purpose (mean:4.65 ± 0.69). They also emphasized that they found the VR application effective in reducing stress (mean:4.58 ± 0.64) (Fig. 4).

When evaluating the side effects of using VR goggles, 61% of participants reported experiencing no side effects. However, 17% reported mild dizziness, 9% experienced loss of balance, 9% mentioned itching or sweating due to contact with the goggles, 4% reported temporary blurred vision, and another 4% reported headache. These side effects did not hinder participants from completing the VR session, and all participants completed the session.

## Discussion

VR technology provides an immersive, multi-sensory, three-dimensional environment that allows users to experience altered reality by creating a sense of ‘presence’[16]. The immersive nature of virtual reality promotes a sense of presence in a virtual environment, which, through controlled visual and acoustic stimulation, is associated with physiological relaxation and stress reduction, similar to the restorative effects of natural environments [17].

This study, which compares the stress levels of emergency physicians following rest and virtual reality use, is among the limited research conducted on healthcare professionals during a shift and represents the first study to specifically investigate the impact of VR on stress levels in an ED setting. Moreover, the ability to compare the effects of both rest and VR use on anxiety levels can be considered a strength of this study. Although no studies have directly examined the effect of rest on the anxiety levels of emergency physicians, a systematic review indicated that while existing evidence suggests a positive trend, it is not possible to draw a definitive conclusion on whether breaks improve physicians' well-being and performance[18]. Although the literature does not provide definitive conclusions on this matter, we considered VR as a comparable stress management method to rest. In our study, we found that VR can effectively reduce anxiety levels during a shift and provides a more significant reduction compared to rest.

Our findings are consistent with previous studies demonstrating the effectiveness of VR in reducing anxiety. In a study conducted by Nijland and colleagues during the COVID-19 pandemic, 10-min VRelax breaks were implemented for intensive care nurses during their shifts, and perceived stress levels were measured before and after VRelax use using the Visual Analog Scale (VAS). The results showed a 39.9% reduction in stress levels among nurses who used VRelax [19]. Our study is similar to this research in terms of using a VR application during shifts, maintaining a comparable session duration, and achieving similar outcomes. However, the VR application we used differed from the one in that study. We selected this particular application because it allows for specific physical activities and provides orientation for using the VR goggles simultaneously during the process. Additionally, while we predefined the intervention time window as 7:00 pm to 10:00 pm, participants were given the flexibility to select the most suitable time within this period. This approach aimed to minimize disruption to the usual shift workflow. In another study from the literature, Beverly et al. presented a three-minute VR simulation featuring a 360-degree nature scene to healthcare professionals working in COVID-19 units, both in direct and indirect patient care roles. They assessed stress levels using the VAS before and after the intervention. The participants’ average stress score, which was 5.5 before the intervention, decreased by 40% to 3.3 following the VR session [20]. Similarly, we found a 36% reduction in anxiety levels in our study following the VR intervention. However, the broad distribution of changes in anxiety levels observed in our study suggests that the effectiveness of VR may vary among individuals. This suggests that VR may not be universally effective and should be applied on an individual basis. On the other hand, the more consistent outcomes observed with rest suggest that it may have a more predictable effect on stress management.

When the subgroups were analyzed, we found that VR reduced stress levels in both male and female participants, as well as in participants with more than two years of experience. However, among participants with two years or less experience although VR reduced stress levels, this reduction was not statistically significant (*p* = 0.15). This may be attributed to the low number of participants in this subgroup. Additionally, physicians in their first few years in the ED often experience a significant learning burden while simultaneously trying to manage occupational stressors. During this period, one of the primary sources of stress may stem from the challenge of adapting to professional demands and acquiring new knowledge, while effective coping mechanisms may not yet be fully developed. These factors might have limited the effectiveness of VR for participants with less than two years of seniority.

The impact of VR on the overall well-being of emergency physicians is noteworthy, particularly in the context of their irregular work schedules. A study by Mazgelytė et al. demonstrated the positive effects of brief VR-based biofeedback-assisted relaxation techniques on psychological, physiological, and biochemical stress indicators [21]. Their research demonstrated that VR-based relaxation techniques reduced salivary steroid hormone levels and increased galvanic skin response values. This suggests that these techniques effectively induced a relaxation state in participants. Consistent with our findings, this evidence supports the potential of VR-based interventions as effective stress management tools.

The First Steps program is an introductory-level VR experience designed to introduce users to VR environments. While it includes some movement that may contribute to stress reduction, its immersive and engaging effects could diminish with repeated use. Given the established benefits of mindfulness and meditation in stress management, VR-based meditation programs may offer a more effective and sustained reduction in stress levels. Integrating structured mindfulness techniques into VR could enhance engagement and provide long-term benefits for stress reduction in emergency physicians.

In this study, in addition to measuring STAI scores, we also gathered participants' feedback and experiences. Overall, we observed that they were highly satisfied with the use and experience of VR. Although some participants felt that the VR session duration was relatively short, it is essential to consider that the ED does not always allow extended breaks.

Future studies should explore different session durations within the constraints of emergency settings while incorporating objective physiological or biochemical markers, such as cortisol levels or heart rate variability, to better evaluate the impact of varying application durations on stress reduction and enhance the reliability and validity of stress and anxiety assessments. Future studies could also benefit from comparing the effects of non-VR breaks, including exercise with VR sessions. Additionally, exploring VR programs incorporating physical activity may further enhance their effectiveness in stress reduction. Additionally, studies involving larger sample sizes and focusing on long-term stress reduction programs incorporating integrated movement or mindfulness components will contribute to the literature by providing more effective strategies for reducing stress and burnout among emergency department physicians.

## Limitations

The study has several limitations. First, the small sample size and the single-center design restrict the generalizability of the findings. Additionally, the stress measurement methods relied on subjective self-reports, which may have been influenced by individual perceptions and a social desirability bias. We assessed state anxiety levels and conducted the second measurements immediately after the break and VR application. Consequently, we could not evaluate the long-term effects on stress levels in the subsequent hours. Furthermore, nearly all participants had no prior experience with VR. The significant reduction in stress levels observed may be attributed to the excitement and curiosity associated with the novelty of the first experience. It remains unclear whether the same effect would be sustained with repeated use. We selected 10 min as the application duration; therefore, the effects of shorter sessions remain unknown. Another limitation involves potential side effects—although no participants in our study discontinued the intervention due to side effects, VR may not be feasible for individuals who experience significant discomfort or adverse reactions to its use. Repeated exposure may diminish the relaxing effects for these individuals. Additionally, due to the limited number of participants experiencing side effects, a detailed analysis of their impact was not feasible, which may limit the generalizability of our findings regarding VR’s safety and side effects. Another limitation is that we could not include participants who wore spectacles in the study. However, this issue could be addressed using VR headsets with adjustable diopters (e.g., HTC Vive XR). Furthermore, while our study primarily focused on reducing anxiety during shifts, we did not assess residents' job performance following the VR intervention.

## Conclusion

According to the results of our study, we found that a 10-min VR intervention during a 24-h shift effectively reduced state anxiety levels among ED residents and proved to be a more effective method than rest. Additionally, we observed a high level of participant satisfaction. Future research should explore the long-term effects of both VR and rest interventions using larger sample sizes, and compare the impacts of various stress management techniques on individuals.

## Supplementary Information

Below is the link to the electronic supplementary material.

Supplementary file1 (MP4 164802 KB)

## Appendix 1. STAI-I form

**Table:**

Self-evaluation questionnaire	1	2	3	4
**1.** I feel calm	( )	( )	( )	( )
**2.** I feel secure	( )	( )	( )	( )
**3.** I am tense	( )	( )	( )	( )
**4.** I feel strained	( )	( )	( )	( )
**5.** I feel at ease	( )	( )	( )	( )
**6.** I feel upset	( )	( )	( )	( )
**7.** I am presently worrying over possible misfortunes	( )	( )	( )	( )
**8.** I feel satisfied	( )	( )	( )	( )
**9.** I feel frightened	( )	( )	( )	( )
**10.** I feel comfortable	( )	( )	( )	( )
**11.** I feel self-confident	( )	( )	( )	( )
**12.** I feel nervous	( )	( )	( )	( )
**13**. I am jittery	( )	( )	( )	( )
**14.** I feel indecisive	( )	( )	( )	( )
**15.** I am relaxed	( )	( )	( )	( )
**16.** I feel content	( )	( )	( )	( )
**17.** I am worried	( )	( )	( )	( )
**18.** I feel confused	( )	( )	( )	( )
**19.** I feel steady	( )	( )	( )	( )
**20.** I feel pleasant	( )	( )	( )	( )

**1:** Not at all, **2: **Somewhat, **3: **Moderately so, **4:** Very much so

## Appendix 2. Survey of user experience and potential side effects of virtual reality headset use

1. Have you ever used VR glasses before?
  ( ) Yes         ( ) No
2. Please rate your experience with the VR glasses based on the following statements
  **Table:**
  	No	1	2	3	4	5	Yes
  VR glasses were easy to use		( )	( )	( )	( )	( )	
  Interaction with the application was realistic		( )	( )	( )	( )	( )	
  I felt like I wasn’t in the hospital		( )	( )	( )	( )	( )	
  I’ve adapted to the VR environment		( )	( )	( )	( )	( )	
  Duration was sufficient		( )	( )	( )	( )	( )	
  VR application was fit for purpose		( )	( )	( )	( )	( )	
  VR application reduced my stress		( )	( )	( )	( )	( )	
3. Did you experience any side effects during or after using VR glasses?
  ( ) Yes        ( ) No
4. Would you like to try VR glasses for the same purpose on your shifts in the future?
  ( ) Yes           ( ) No
5. Have you felt any side effects during or after using VR glasses? *(you can select more than one)*
  **Table:**
  ⎕	Headache
  ⎕	Dizziness
  ⎕	Nausea or vomiting
  ⎕	Loss of balance
  ⎕	Other (*Please specify: *_________________________)
